# Effects of Shenque Moxibustion on Behavioral Changes and Brain Oxidative State in Apolipoprotein E-Deficient Mice

**DOI:** 10.1155/2015/804804

**Published:** 2015-02-22

**Authors:** Juntian Liu, Baixiao Zhao, Yingxue Cui, Yuhai Huang, Chang Huang, Jian Huang, Li Han, Lixing Lao

**Affiliations:** ^1^School of Acupuncture and Moxibustion and Tuina, Beijing University of Chinese Medicine, Beijing 100029, China; ^2^Beijing Hospital of TCM, Beijing 100029, China; ^3^School of Chinese Medicine, The University of Hong Kong, Hong Kong

## Abstract

*Purpose*. To determine whether moxibustion influences the learning and memory behavior of ApoE−/− male mice, and investigate the mechanism of moxibustion on the alteration of oxidized proteins (glial fibrillary acidic protein, *β*-amyloid) in hippocampus. *Methods*. Thirty-three ApoE−/− mice were randomly divided into 3 groups (*n* = 11/group): moxibustion, sham moxibustion, and no treatment control. Wild-type C57BL/6 mice (*n* = 13) were used for normal control. Moxibustion was performed with Shenque (RN8) moxibustion for 20 minutes per day, 6 days/week for 12 weeks. In sham control, the procedure was similar except burning of the moxa stick. Behavioral tests (step-down test and Morris water maze task) were conducted in the 13th week. The mice were then sacrificed and the tissues were harvested for immune-histochemical staining. *Results*. In the step-down test, the moxibustion group had shorter reaction time in training record and committed less mistakes compared to sham control. In immune-histochemical study, the moxibustion group expressed lower level of GFAP and less aggregation of *β*-amyloid in the hippocampus than the sham control. *Conclusion*. Our findings suggest that moxibustion may enhance learning capability of ApoE−/− mice. The mechanism may be via inhibiting oxidized proteins (GFAP and *β*-amyloid) in astrocytes.

## 1. Introduction

Alzheimer disease (AD) which is regarded as highly likely outcome of dementia patients is due to complex pathological causes such as oxidative stress, cell cycle aberration, transition metal dyshomeostasis, neurofibrillary tangle (NFT) formation, and *β*-amyloid (Ab) oligomerization/fibrillation [1]. Several genes have been proved to be highly activated in brains with AD, including AEP (activation of asparaginyl endopeptidase leading to hyperphosphorylation), APP, PS1, and apolipoprotein E (ApoE). ApoE is a main gene for transportation of lipid and lipoprotein in plasma and neurons. Thus ApoE is regarded as a crucial role in amyloid clearance and/or metabolism [2]. As reported, ApoE can be encoded into 3 alleles (i.e., ƹ2, ƹ3, and ƹ4), among which the ƹ4 is believed to be a key risk factor to AD, while the ƹ2 serves a role of neuron protection [3]. Several studies have shown that ApoE4 targeted-replacement mice expressed increasing p-eIF2/eIF2 levels in brains with impairment of learning function [4] or anxiety which directly leads to decrease of microtubule-associated protein in central nucleus [5]. ApoE4 can also affect the cerebrovascular system by accumulation of neurotoxins such as *β*-amyloid (A*β*) [6] and activate expression of GFAP and other 5 oxidized proteins which were already discovered in AD patients' brains [7, 8]. Therefore, ApoE−/− mice are widely used as AD animal model.

Several behavioral tests, such as classic Morris water maze task and step-down test, are frequently used in researches on AD. These tests are designed to assess the spatial learning and memory that can be influenced by several factors including the characteristics of animals (species, strains, sex, and age) and the differences of training procedure. The function of hippocampus can be demonstrated by these tests.

As part of traditional acupuncture practice, moxibustion applies the heat of burning moxa floss, primarily from* Artemisia vulgaris*, to stimulate specific spots, known as acupuncture points on the surface of skin [9]. For thousands of years, moxibustion has been widely used in clinical and family health care in China and other Asian countries. It works through not only direct or indirect thermal stimulation at various temperature levels but also chemical intervention of moxa smoke. Recently, moxibustion has been used for treatment of several senile diseases, such as osteoarthritis [10] and depression [11], although few researches focus on the underlying mechanism. We hypothesized that moxibustion had an effect on improving learning and memory and its mechanism was associated with inhibition of GFAP, attenuation of *β*-amyloid, activation of NADPH, and decrease of oxidative stress, which in turn protected the neuron.

## 2. Materials and Methods

### 2.1. Animal Preparation

All procedures of the animal experiments strictly complied with the World Health Organization's International Guiding Principles for Biomedical Research Involving Animals and were approved by the local ethics committee of Beijing University of Chinese Medicine.

Considering the mortality of mice, we purchased 13 with same genetic background C57/BL6 mice and 33 genetically knocked-out ApoE mice from the Animal Center of the Medical College, Peking University. This study used male mice of 8 weeks of age and weighing around 27–29 g. All animals were individually housed at the constant temperature (18°C–23°C), with light from 6 a.m. to 6 p.m. and humidity of 50%–60%. Only ApoE−/− mice were fed with high-fat diet; C57/BL6 mice were fed with regular diet all throughout the experiment duration. Recent studies reported that high-fat diet plays a support role in ApoE4 causing AD. According to the researches made by Park et al. [12] and Ping et al. [13], ApoE−/− mice fed with high-fat diet would accelerate AD-like pathologies and cognitive deficits by increasing A*β*, p-tau, mortalin levels, and microglial activation, decreasing the amount of Nissl bodies in front lobe. Therefore we took ApoE−/− mice with high-fat diet as AD models.

### 2.2. Experiment Design

Thirty-three ApoE−/− mice were randomly divided into three different groups. The groups for our experiments included no treatment control group, sham moxibustion group, and moxibustion group (11 for each group). Meanwhile, 13 C57BL/6 normal mice served as normal control group.

**Figure 1:**
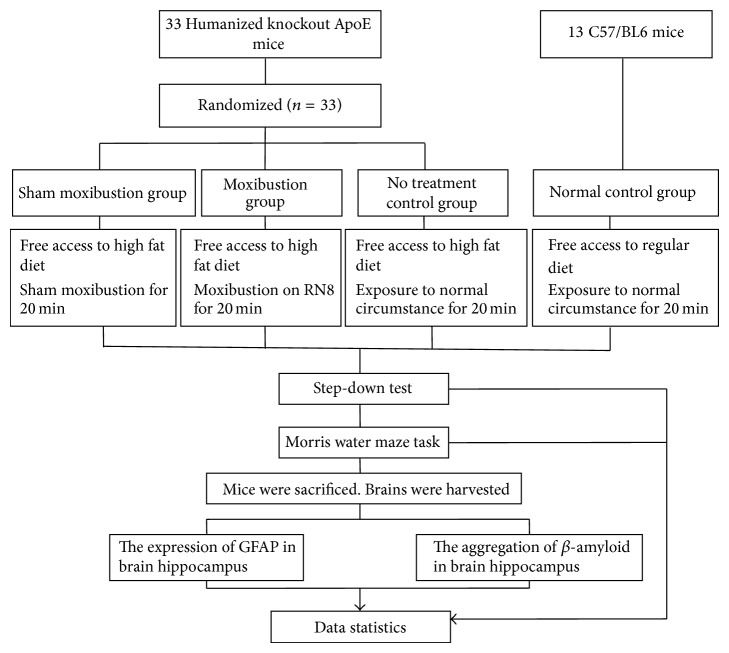
Experimental procedures.

The moxibustion procedure started on the 8th day after 1-week adaption for all mice. Mice of moxibustion group received 20 min of moxibustion per day, 6 days per week for 12 weeks on Shenque (RN8). The behavioral tests were given in the 13th week, 1 week after the completion of treatment. Brain GFAP level and area of *β*-amyloid deposits were evaluated by immune-histochemical staining and Congo red staining, respectively (Figure 1).

### 2.3. Moxibustion Treatment

In our experiment, a plastic fixator with a hole on the bottom was used to fasten the mouse. A mouse was put into a fixator with its umbilicus exposed from the hole. The operator held the fixator and squeezed its tail with fingers to hold a pose with one hand and exerted indirect moxibustion over Shenque (RN8) with the other hand. The diameter of moxa stick was 5 mm; the distance between umbilicus and the ignited tip of the moxa stick was approximately 2-3 cm so that the temperature of moxibustion was kept in a proper range. Moxibustion group was manipulated in room 1.

In sham moxibustion group, all measures were similar to those in moxibustion group except that the moxa sticks were not ignited and then there was no moxa smoke in air. Mice of sham moxibustion group were settled in room 2. The meaning of sham moxibustion is to evaluate the effect of moxibustion.

**Figure 2:**
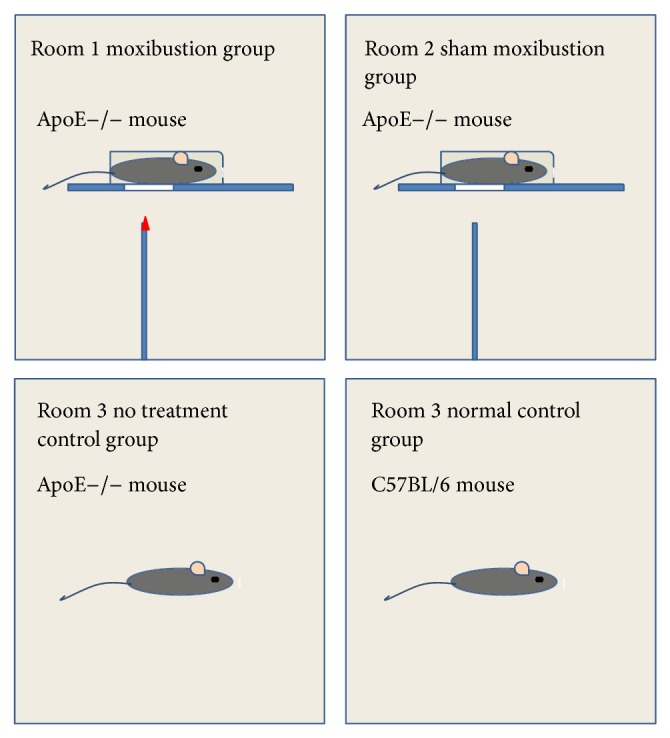
Intervening measures.

Mice in normal and no treatment control groups were exposed without manipulations in room 3 free of moxa smoke (Figure 2).

### 2.4. Behavioral Test

#### 2.4.1. Step-Down Test

The step-down test [14, 15] was carried out in 2 days including training and retention sessions. The apparatus consisted of a transparent box (20 cm × 20 cm × 60 cm) featuring a stainless-steel grid floor and a robber platform (diameter 10 cm, height 4.5 cm) fixed in the centre of the box. Electric shocks (36 v) were delivered to the grid floor with a stimulator. Each time mouse steps down the platform it would be punished by electric shock. At the beginning of training session, mice were allowed to adapt for 3 min in the box. After 3 min, electric shocks were delivered and mice jumped on the platform to avoid stimulation; the latency to reach the platform and the number of mistakes made in 5 min were recorded. A mistake was counted whenever the mouse stepped down the platform and touched the floor in electric mode. Retention session was carried out 24 hours after training session. The mice were again placed on the platform, and the latency to step down on the grid for the first time and the number of mistakes within 5 min were measured as learning performances.

#### 2.4.2. Morris Water Maze Task

In the 13th week, mice were tested for spatial learning and memory functions in water maze [16] for 5 days. A circle pool (diameter 140 cm, height 50 cm) of opaque water was divided into 4 quadrants mixed with milk at constant temperature (23°C). There were 4 different visual cues hang in each quadrant separately. A plexiglass platform (diameter 6 cm) was randomly placed in one quadrant 5 mm under the water level.

On the first day, mice were first given 2 min of adaption to swimming in the pool without platform and then received four 60 s learning sessions with platform and 12 min resting period between each learning session. Each mouse was put into the water facing the side of the pool in 4 quadrants. In the next 3 days, mice were only trained to locate the hidden platform. When mice failed to find the platform, they would be led to the platform and rested for 60 s. Each mouse would get 4 sets of escape latency data based on its performance in 4 entry points every day. Then the average of these data would indicate the spatial learning latency of mice.

On the fifth day, when learning session completed, the platform was removed from the pool. The spatial memory of mice, including the escape latency in the quadrant with absence of platform, and swimming latency in other quadrants were recorded within 60 s. The escape latency in percentage of 60 s would reveal the spatial memory of mice.

### 2.5. Immunohistochemical Staining

In the 14th week, following the behavioral test, the mice were deeply anesthetized with pentobarbital (300 *μ*g/kg body weight); then the brains were harvested on an ice board and fixed in a bottle with 4% paraformaldehyde solution for 24 hours at room temperature. Brains were embedded in wax and then cut into sagittal slices of 50 *μ*m thickness by vibratome (microslicer model 3000, Pelco International) [17]. Afterwards, sections of every group were dewaxed and rehydrated. Next, sections were boiled in 0.01 mol citric acid buffer for 2.5 min. After cooling, these sections were incubated in 3% hydrogen peroxide for 8 min and then washed with water and PBS. The incubation was applied by the primary antibody (anti-GFAP raised in rabbit, Abcam, 1.0 *μ*g/mL) overnight at room temperature. Then sections were washed in fresh PBS 3 times for 10 min each before incubating with the secondary antibody (GFAP: anti-rabbit Texas Red raised in donkey, Jackson, 15 *μ*g/mL) for 30 min at room temperature. Finally sections were dyed with diaminobenzidine and haematoxylin, respectively, mounted on slides, dehydrated, and cover-slipped with DPX Mountant (Sigma-Aldrich) and imaged with a Confocal Microscope (Olympus Spinning Disk Confocal, Center Valley, PA, USA) [18].

Same as the immunohistochemical staining, sections were dewaxed and rehydrated before staining with Congo red for 4 hours at room temperature, then washed with saturated NaCl, dehydrated (80% ethanol, 95% ethanol, and 100% ethanol), and cover-slipped with DPX Mountant and allowed to dry overnight [19]; amyloid plaque would exhibit red on brain tissue.

### 2.6. Statistic Analysis

Data of Morris water maze, part of step-down test, average optical density of GFAP, and area of Congo staining for A*β* were collected and analyzed by analysis of variance (ANOVA). Post hoc analyses were conducted using the Student-Newman-Keuls test. One-way ANOVAs were conducted to analyze significant interactions among different groups. These values were reported as means ± standard error, and *P* < 0.05 was considered to be statistically significant. An independent sample nonparametric test (Mann-Whitney *U* Test) was used for retention latency and mistakes of step-down test to compare data from different groups. All data were analyzed with spss19.0.

The sample detection and data statistic were undertaken in condition of blind to grouping.

## 3. Results

### 3.1. Behavioral Test

#### 3.1.1. Step-Down Test

In step-down test, the moxibustion group demonstrated shorter training time (*P* < 0.05) and committed fewer mistakes (*P* < 0.05) than sham moxibustion group. On the other hand, there was a decreasing tendency of reaction time and mistakes in moxibustion group compared with the sham moxibustion group in terms of retention record (Table 1).

#### 3.1.2. Morris Water Maze Task

During visible platform training, the platform was clearly set in training quadrant and all mice improved their performance through training. There was no benefit from moxibustion on latency to locate the platform compared with sham moxibustion group. Meanwhile, the no treatment control group required more time to locate the platform (Figure 2).

**Figure 3:**
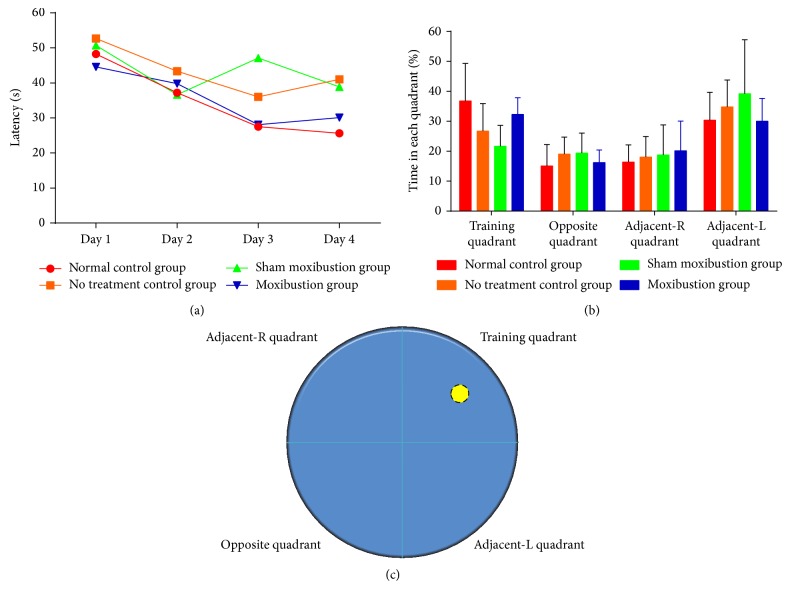
The performance of 4 groups in Morris water maze task. (a) Each point represents group mean latency (s) to reach the hidden platform in four groups during visible platform training. (b) Bar chart shows the percentage of time spent in each quadrant within 60 s during absence platform spatial memory test. (c) The Morris water maze pool is divided into 4 quadrants. The yellow circle represents the platform which is placed in training quadrant.

No significant difference was found among the groups. After 4 days of spatial learning, the memory retention of mice was recorded as the time searching for absence platform in training quadrant in percentage of 60 s. All groups except the no treatment control manifested longer retention in training quadrant, more than the total length of 25%. Among all these 4 quadrants, all groups except for no treatment control tended to stay longer in training quadrant rather than opposite and adjacent-R quadrants (Figure 3).

### 3.2. Immunochemistry Straining

#### 3.2.1. Glial Fibrillary Acidic Protein

**Figure 4:**
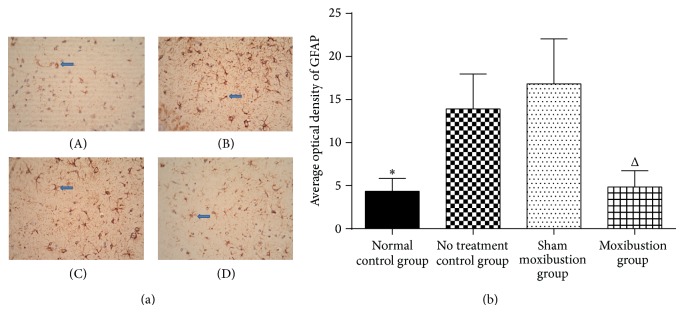
Hippocampus glial fibrillary acidic protein. (a) The pictures reflect the expression of GFAP in 4 groups (original magnification ×400). (A) represents normal control group (4.33 ± 1.52), (B) represents no treatment control group (13.90 ± 4.06), (C) represents sham moxibustion group (16.82 ± 5.23), and (D) represents moxibustion group (4.85 ± 1.89). Blue arrows identify examples of clusters of GFAP positive cells. (b) Bar graph represents GFAP immunoreactivity in the hippocampus CA3. ^*^ *P* < 0.05 versus no treatment control group; ^Δ^ *P* < 0.05 versus sham moxibustion group.

The optical density of GFAP in hippocampus expressed the highest level in no treatment control group, came second in sham moxibustion group, and was the least in moxibustion and normal control groups. Compared with normal control group and no treatment control group, moxibustion group and sham moxibustion group both showed significant differences (Figure 4).

#### 3.2.2. *β*-Amyloid Plaque of Hippocampus

**Figure 5:**
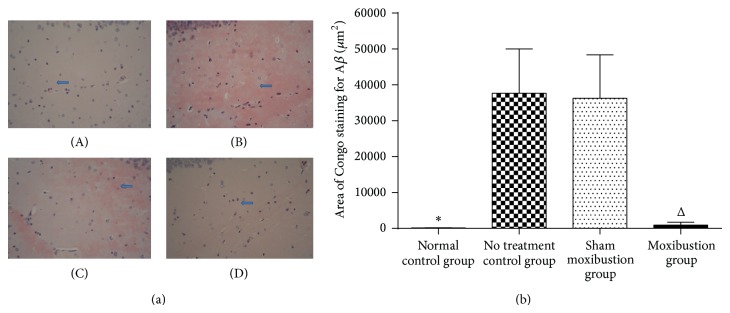
Hippocampus *β*-amyloid plaque. (a) The pictures reflect the *β*-amyloid plaque expression in 4 groups under Congo red straining (original magnification ×400). (A) represents normal control group (101.25 ± 82.68), (B) represents no treatment control group (37661.70 ± 12349.53), (C) represents sham moxibustion group (36281.30 ± 12104.97), and (D) represents moxibustion group (947.70 ± 768.70). *β*-Amyloid appears as a cloud mass and shows strongly Congo red positive (B, C). (b) The graph shows different levels of *β*-amyloid; thus it can be seen that normal control group got less area of Congo staining for *β*-amyloid than that of no treatment control group. Same situation occurs between sham moxibustion group and moxibustion group. ^*^ *P* < 0.05 versus no treatment control group; ^Δ^ *P* < 0.05 versus sham moxibustion group.

Moxibustion group showed a remarkable decrease of *β*-amyloid plaque level compared with sham moxibustion group. No treatment control group developed higher amyloid plaque level than normal control group, which proved the existence of significant difference (Figure 5).

## 4. Discussion

In this study, we explored the antiaging function of moxibustion for ApoE−/− mice and discovered that moxibustion could effectively improve the cognitive behavior through step-down test and Morris water maze. Meanwhile we confirmed that ApoE−/− mice of 5 months have defects in cognitive function. This phenomenon had been observed by Masliah et al. since 1997. Additional researches have provided evidence on the fact that adult ApoE−/− mice exhibited progressively worsening cognitive dysfunction with age [20]. They also demonstrated that ApoE4 mice showed hippocampus-dependent learning deficits in extremely early stages of life [4]. Possible reasons were brain short of acetylcholine and abnormal accumulation of *β*-amyloid [21] both combined intimately with knocking out of gene ApoE.

Interestingly, in behavioral test, we observed that moxibustion group only performed better in training section but not in retention section. Similar to step-down test, there was a trend toward less spatial learning retention of Morris water maze task in moxibustion group than sham moxibustion group, which indicated that moxibustion might only enhance learning capability. We hypothesized that spatial learning and memory have different mechanisms in brain. Spatial learning and memory process depended on the integrity and functions of certain brain regions such as hippocampus [22]. Only damaging one-third of the dorsal part of hippocampus would cause several spatial memory defects [23]. The spatial learning was more vulnerable to chronic neuron death in CA1 of hippocampus [24]. In addition, we suspected that the enhancing of learning was not premised on the improvement of memory. Unlike spatial learning capability, memory was less affected by repeated training. Hence it might serve as a long term target which applied to evaluating the degree of disturbing animals' long term behavior.

Aging will lead to several neuropathology processes. Glial fibrillary acidic protein, one of the cytoskeletal intermediate filaments of astrocytes in central nervous system, is a typical astrocyte marker of increasing expression in brain with age both in animals [18, 25] and in humans [26]. Recent study on proteomic identification of oxidized proteins in brain of ApoE−/− mice showed that old wild mice and ApoE−/− mice got higher GFAP expression revealing close relationship between AD and ApoE−/− mice [27]. AD is characterized by intracellular fibril deposition. Deposition of *β*-amyloid resulted from facilitating misfolding of amyloid-precursor protein [28]. As a matter of fact, *β*-amyloid is a notable marker in AD diagnosis, which is also involved in disease progression. Since several serious neural system disorders including dementia and AD in the elderly are mainly caused by cerebral amyloid angiopathy (CAA) via reduction of blood flow [29], ApoE in lipidation state may contribute to critical impact on A*β* fibril formation [30] and finally result in neural inflammatory responses such as synaptic dysfunction, neuronal death, and neurodegeneration.

The overexpression of GFAP and *β*-amyloid further demonstrated that ApoE−/− mice could imitate not only clinical symptoms but also pathogenic changes of AD. As expected, GFAP immune-reactivity was significantly increased in hippocampal region CA3 of no treatment control group. Part of the explanation for age-dependent increase of GFAP consisted in oxidative stress, which suggested that GFAP plays an important role in astrocyte protection [27]. Nevertheless overexpression of GFAP also can be lethal and lead to the discovery of Alexander disease, a severe neurodegenerative disorder [31]. In addition the age-dependent increase of GFAP may contribute to inefficient or defective cell division in neural stem cells [32, 33]. As a consequence, maintaining appropriate active level of GFAP will have a critical influence in aging process and general neuropathology [28].

**Figure 6:**
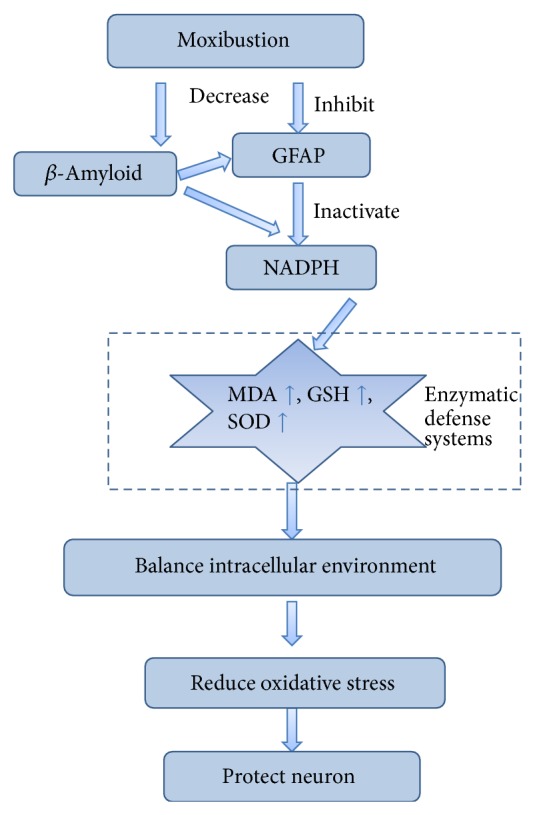
Targeting the NADPH by evoking oxidative stress in astrocytes.

The decreased content of these 2 proteins in hippocampus intervened by moxibustion might provide evidence on moxibustion's antiaging function. In vitro trials demonstrated that *β*-amyloid could activate GFAP expression in astrocytes via several mechanisms [34]. We confirmed that moxibustion can activate NADPH, triggering the oxidative stress which results in neuronal death [35]. Our data showed reduced GFAP in hippocampus of ApoE−/− mice after moxibustion, exhibiting lower oxidant stress level and less astrocyte impairment in brain, and directly inducing better performances in behavioral tests (Figure 6). As shown, ApoE−/− mice exhibited high level of *β*-amyloid deposition, while the treatment of moxibustion could change this pathological process to some degree. The possible explanation could be that *β*-amyloid deposition is a dynamic and reversible process, whose potential impact factors include environment and the specific protein itself.

There are some limitations in our study. Based on the clinical observations, we designed the treatment time as 3 months. We did not evaluate the result after 4~8 weeks to follow-up. Thus we cannot fully understand the therapeutic effect of moxibustion for AD. As a matter of fact, we are considering the addition of different observation points during the whole treatment. We will extend the follow-up evaluation to weeks 4 and 8 in our future studies.

Nevertheless, the results of our study suggest that moxibustion could change learning capability of ApoE−/− mice by means of decreasing the GFAP and *β*-amyloid deposition in the hippocampus. It indicates that moxibustion might have antiaging function by changing the oxidant state in central nervous system.

## Figures and Tables

**Table 1:** Latency to reach the insulative platform and times of jumping out of the platform.

	Normal control group (*n* = 13)	No treatment control group (*n* = 6)	Moxibustion group (*n* = 9)	Sham moxibustion group (*n* = 6)
Training latency^a^	5.77 ± 4.96^*^	74.68 ± 55.95	4.93 ± 2.54^△^	71.02 ± 84.51
Training mistakes^a^	3.69 ± 1.70	3.5 ± 2.59	0.33 ± 0.50^△^	3.67 ± 2.34
Retention latency^b^	118.50(122.55)^*^	2.55 (37.8)	180 (14.5)	180 (51.525)
Retention mistakes^b^	1(1)^*^	0 (0.5)	0 (0.5)	0 (1)

^a^Mean ± standard deviation; ^b^median (interquartile range). ^*^ *P* < 0.05 versus the no treatment control group; ^△^ *P* < 0.05 versus sham moxibustion group.
